# hCLOCK Causes Rho-Kinase-Mediated Endothelial Dysfunction and NF-*κ*B-Mediated Inflammatory Responses

**DOI:** 10.1155/2015/671839

**Published:** 2015-10-25

**Authors:** Xiao Tang, Daqiao Guo, Changpo Lin, Zhenyu Shi, Ruizhe Qian, Weiguo Fu, Jianjun Liu, Xu Li, Longhua Fan

**Affiliations:** ^1^Institute of Vascular Surgery, Department of Vascular Surgery, Zhongshan Hospital, Fudan University, Shanghai 200032, China; ^2^Department of Physiology and Pathophysiology, Fudan University Shanghai Medical College, Shanghai 200032, China; ^3^Department of Vascular Surgery, Qingpu Branch of Zhongshan Hospital, Fudan University, Shanghai 200032, China

## Abstract

*Background*. The human Circadian Locomotor Output Cycle protein Kaput (CLOCK) gene was originally discovered as a regulator of essential human daily rhythms. This seemingly innocuous gene was then found to be associated with a multitude of human malignancies, via several biochemical pathways. We aimed to further investigate the role of hCLOCK in the hypoxia-oxidative stress response system at the biochemical level. *Methods*. Expression levels of Rho GTPases were measured in normoxic and hypoxic states. The effect of hCLOCK on the hypoxic response was evaluated with the use of a retroviral shRNA vector system, a Rho inhibitor, and a ROS scavenger by analyzing expression levels of hCLOCK, Rho GTPases, and NF-*κ*B pathway effectors. Finally, in vitro ROS production and tube formation in HUVECs were assessed. *Results*. Hypoxia induces ROS production via hCLOCK. hCLOCK activates the RhoA and NF-*κ*B signaling pathways. Conversely, inhibition of hCLOCK deactivates these pathways. Furthermore, inhibition of RhoA or decreased levels of ROS attenuate these pathways, but inhibition of RhoA does not lead to decreased levels of ROS. Overall findings show that hypoxia increases the expression of hCLOCK, which leads to ROS production, which then activates the RhoA and NF-*κ*B pathways. *Conclusion*. Our findings suggest that hypoxic states induce vascular oxidative damage and inflammation via hCLOCK-mediated production of ROS, with subsequent activation of the RhoA and NF-*κ*B pathways.

## 1. Introduction

The human Circadian Locomotor Output Cycle protein Kaput (CLOCK) gene was originally discovered as a regulator of essential human daily rhythms, as its name implies. The human circadian rhythm encompasses a multitude of physiologic activities, from macroscopic processes such as sleep-wake cycles and core body temperature to molecular mechanisms such as hormone secretion, metabolism, and cell cycle timing [1, 2]. These are all ultimately controlled by a system of feedback loops, with one well-established model describing interactions between the heterodimer transcriptional factors CLOCK and BMAL1, the cryptochromes Cry1 and Cry2, and the period regulator genes Per1, Per2, and Per3 [3]. Of these, CLOCK has been described as the master controller gene. On the other hand, however, some studies have shown that the CLOCK protein is not required for rhythmic gene expression but attributes to robust maximal peak expression levels of rhythmic gene [4–6].

The sphere of influence of CLOCK extends further still, as disruptions in circadian rhythm and defects in circadian rhythm genes are known to be associated with human malignancy. Recent research has described the proneoplastic effects of hCLOCK in brain, breast, colorectal, renal, hepatocellular, and endometrial cancer [7–11]. Despite its seemingly innocent name, hCLOCK operates via multiple pathways related to the regulation of apoptosis, cell proliferation, hormone receptor expression, and hypoxia response.

The hypoxia response in cancer progression has been previously described but has not been studied with particular attention to CLOCK [12]. Broadly speaking, cells respond to hypoxia, pseudohypoxia, or gene mutations by generating reactive oxygen species (ROS) which cause oxidative damage to DNA. Some of these resultant DNA modifications are involved in the initiation of various cancers [13, 14]. Interestingly, cancer cells themselves must express increased antioxidant protein levels to protect against these same ROS [15]. This introduces a delicate balance between ROS and antioxidant levels that cancer cells need to precisely manage in order to develop and survive. Antineoplastic therapeutic strategies must therefore be tailored accordingly. Additional study of the relationships between hCLOCK and oxidative damage and the inflammatory response is required.

Research over the past decade has discovered new molecular links between the Rho GTPases and the nuclear factor *κ*B (NF-*κ*B) pathway in inflammation [16]. The Rho GTPases are a collection of 20 proteins, most notably RhoA, Rac, and Cdc42, which are widely found within cells and serve to mediate cell spreading, adhesion, and movement. ROCK1 is a major downstream effector of the Rho GTPases, in particular RhoA. These Rho GTPases are regulated by GTPase-activating proteins (GAPs), guanine-nucleotide-exchange factors (GEFs), and guanosine-nucleotide-dissociation inhibitors (GDIs). The NF-*κ*B pathway is a signaling cascade involved in multiple physiologic processes, most notably its close integration with tumor suppressor pathways, its proinflammatory functions, chronic inflammatory, and its role in immune homeostasis [17–20]. Inducers of the NF-*κ*B pathway, such as tumor necrosis factor (TNF), interleukins (IL), and viral and bacterial products such as lipopolysaccharide (LPS), have been shown to induce Toll-like receptor (TLR) signaling and cellular stress, such as DNA damage and hypoxia [19]. Activation of the NF-*κ*B pathway by Rho GTPases was found to increase expression of inflammatory mediators IL-1, collagenase-1 [21], and tumor necrosis factor alpha (TNF-*α*) [22] and to produce a mutant version of the inflammatory suppressor p120 catenin [23]. Rho GTPases have been shown to inhibit the NF-*κ*B pathways in certain situations as well, when activated by lipopolysaccharide (LPS) and interferon gamma (IFN-*γ*), disrupting the precise homeostasis of central nervous system inflammation [24].

The intersection between the inflammatory and neoplastic mechanisms of hCLOCK and Rho GTPase/NF-*κ*B therefore carries important potential for application toward future therapeutic strategies. We aimed to further investigate the role of hCLOCK in the hypoxia response and oxidative stress at the biochemical level.

## 2. Materials and Methods

### 2.1. Chemicals

A Rho inhibitor allows specific study of this Rho pathway and uncovers alternate pathways of ROS production. A ROS scavenger allows facile manipulation of ROS concentrations. Cell-permeative Rho inhibitor C-3 transferase (CT-04) was purchased from Cytoskeleton (Denver, CO, USA) and dissolved in DMSO to make a 20 *μ*g/mL stock solution. Cells were treated with dilutions of this CT-04 solution for 24 hours prior to analysis. Tiron (4,5-dihydroxy-1,3-benzene disulfonic acid-disodium salt, Sigma, St. Louis, MO, USA), a ROS scavenger, was dissolved in dimethyl sulfoxide (DMSO, final concentration less than 0.1%) to make a 100 mM stock solution. Cells were treated with dilutions of this Tiron solution for 12 hours prior to analysis.

### 2.2. Cell Culture and Treatment

Human Umbilical Vein Endothelial Cells (HUVECs) were purchased from the American Type Culture Collection (ATCC, Rockville, MD, USA) and maintained in an EGM-2 BulletKit (Lonza, Basel, Switzerland) containing 10% fetal bovine serum, 100 U/mL penicillin, and 100 *μ*g/mL streptomycin.

The Xvivo Closed Incubation System (Xvivo system 300 C, BioSpherix, Lacona, New York, USA) was used in order to accurately maintain different oxygen tensions in different chambers. Cells were grown at 37°C in an atmosphere of 5% CO_2_/95% room air. After 24 hours of cultivation in the conventional cell culture, the cells were divided into separate chambers with different oxygen controls for varying periods of time and then harvested for measurement of ROS level and tube formation, as well as Western blotting.

### 2.3. Preparation of the Retroviral Vector

Retroviruses were used to introduce hCLOCK into the HUVECs and serve as vectors for negative control cells and scrambled control cells.

Stable transfectants overexpressing hCLOCK (GenBank Accession Number: NM_004898) were generated via retroviral transduction using a pGV186 retroviral vector (GeneChem Co., Ltd., Shanghai, China). As a control, a retroviral vector expressing green fluorescent protein alone was also generated.

The short hairpin RNA (shRNA) sequences targeting hCLOCK were constructed using a pGV113 retroviral vector (GeneChem Co., Ltd., Shanghai, China). Scrambled shRNA expressing vectors (SCR) serving as controls were made in a similar fashion.

### 2.4. Determination of RhoA Activation

RhoA activation was assessed by loading the RhoA protein in cell lysates, using the RhoA Activation Assay Biochem Kit (Cytoskeleton, Denver, CO, USA), based on the method described by Cao et al. [25], whereby cellular GTP-bound RhoA is detected by Western blot after affinity precipitation with a fusion protein containing glutathione-S-transferase (GST) and the Rho-binding domain of Rhotekin (GST-RBD). Briefly, total cellular proteins were extracted and quantitated with a Bradford protein assay (Bio-Rad, Hercules, CA, USA). These proteins were then incubated with 25 *μ*g aliquots of brightly colored glutathione affinity beads coupled to GST-RBD in order to pull down the GTP-bound form of RhoA. RhoA activation was quantitatively analyzed by standard Western blot analysis using anti-RhoA antibody.

### 2.5. Measurement of Reactive Oxygen Species (ROS) Levels

Reactive oxygen species (ROS) is a term encompassing a variety of reactive molecules and free radicals derived from molecular oxygen. ROS generation in HUVECs was evaluated using the oxidant-sensing 2′,7′-dichlorofluorescein diacetate (DCFH-DA, 5 *μ*M, Invitrogen, Grand Island, NY, USA). DCFH-DA is a nonpolar nonfluorescent dye which is converted into the polar, highly fluorescent DCF by cellular esterases in a dose-dependent manner when oxidized by intracellular ROS. The fluorescence intensity of DCFH was measured using a spectrophotometer (Leica, Heidelberg, Germany) at an excitation wavelength of 488 nm and an emission wavelength of 525 nm.

### 2.6. Tube-Formation Assay

Tube formation in HUVECs is a well-established in vitro assay of angiogenesis reorganization. Compounds inhibiting tube formation could be useful in various inflammatory or neoplastic disease states. Tube formation was assessed in HUVECs among the control normoxic group and the hypoxic scrambled control (SCR) and shCLOCK-transduced groups. HUVECs (2 × 10^4^ per well) were transduced with pGV113-scrambled control (SCR) or pGV113-shRNA-hCLOCK (shCLOCK) and seeded into Matrigel-coated wells of a 24-well plate. Photographs were taken with a Leica DFC290 digital microscope camera (Leica Camera AG, Wetzlar, Germany) 8 hours later.

### 2.7. Western Blot Analysis

Relative expression levels of hCLOCK, RhoA, Rac1, IL-6, ROCK1, COX2, Phospho-NF-*κ*B p65, and *β*-actin were measured by Western blotting using standard methods. Anti-Rac1 (ab15880) and anti-hCLOCK (ab98948) antibodies were purchased from Abcam (Cambridge, MA, USA). Anti-IL-6 antibody (21865-1-AP) was purchased from Proteintech (Chicago, IL, USA). Anti-ROCK1 (# 4035), anti-COX2 (# 4842), and anti-Phospho-NF-*κ*B p65 (Ser536, # 3033) antibodies were purchased from Cellsignal (Beverly, MA, USA). Anti-*β*-actin antibody (sc-47778) was purchased from Santa Cruz Biotechnology (Santa Cruz, CA, USA). Band intensities of RhoA were normalized to the band intensities of the total RhoA. The band intensities of the other proteins were normalized to the band intensity of the cell structural protein *β*-actin.

### 2.8. Statistical Analysis

All results reported represent the mean ± SD of at least three experiments performed in triplicate. Statistical comparisons between groups were made using two-tailed *t*-tests comparing two variables. Differences were considered statistically significant if *p* < 0.05.

## 3. Results

### 3.1. Expression Levels of hCLOCK and RhoA Are Increased in a Hypoxic State

**Figure 1:**
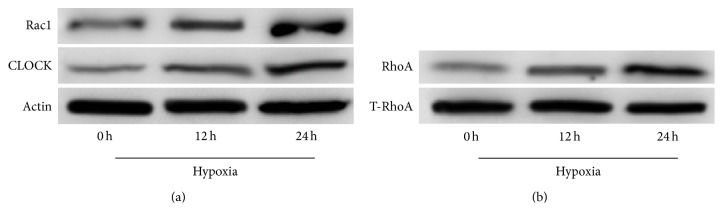
Effect of hypoxia over time on protein expression levels of hCLOCK and RhoA in HUVECs. (a) Western blot analysis was performed to evaluate Rac1 and CLOCK protein expression levels in HUVECs exposed to a hypoxic environment. Expression levels are measured at 0, 12, and 24 hours, normalized to *β*-actin control. (b) RhoA activation assay and Western blot analysis were performed to measure protein expression levels of activated GTPase-bound RhoA in HUVECs exposed to a hypoxic environment. Expression levels are measured at 0, 12, and 24 hours, normalized to total cellular RhoA.

We measured the effects of a hypoxic state on expression levels of hCLOCK and the Rho GTPases RhoA and Rac1 in HUVECs via Western blot over a 24-hour period. Relative levels of both Rac1 and hCLOCK were found to increase significantly at both the 12-hour and 24-hour time points in hypoxic environments (Figure 1(a)). Furthermore, levels of RhoA GTPase increased with time exposed to hypoxia as well, when normalized to total RhoA (T-RhoA) (Figure 1(b)). These findings demonstrate that hCLOCK and RhoA are both involved in the hypoxic response and form the fundamental basis of the remainder of this study.

### 3.2. Hypoxia Induces ROS Production via hCLOCK and Inhibits HUVEC Tube Formation

**Figure 2:**
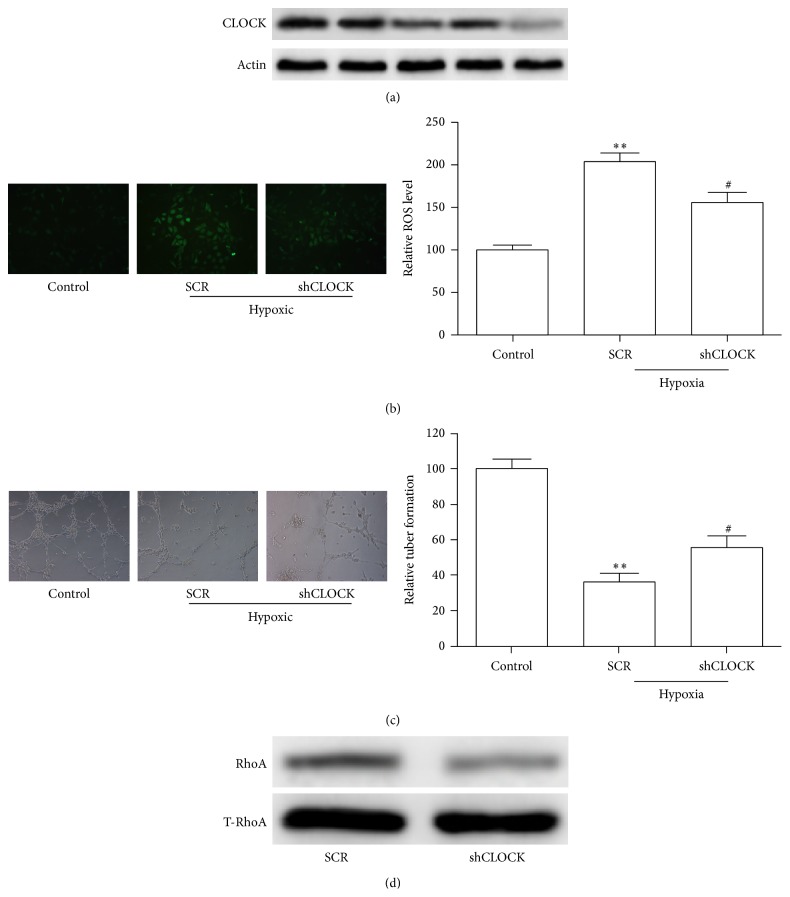
Effect of knockdown of hCLOCK on ROS production, tube formation, and RhoA activity. (a) Western blot analysis was performed to confirm successful knockdown of hCLOCK. (b) Representative images obtained during analysis of fluorescence intensity, comparing ROS levels with and without knockdown of hCLOCK. The control HUVEC group was kept in normoxic conditions and was transduced with a control retroviral vector. The SCR HUVEC group was exposed to hypoxic conditions and was transduced with a scrambled control retroviral vector. The shCLOCK HUVEC group was exposed to hypoxic conditions and transduced with an hCLOCK knockdown retroviral vector. Bar graph shows relative ROS levels normalized to the normoxic control group. ^*∗∗*^ *p* < 0.01 compared to control; ^#^ *p* < 0.05 compared to SCR. (c) Photographs obtained during tube-formation assay comparing tube-formation levels in HUVECs with and without knockdown of hCLOCK. Bar graph quantifies relative tube-formation levels. ^*∗∗*^ *p* < 0.01 compared to control; ^#^ *p* < 0.05 compared to SCR. (d) Western blot analysis showing activated RhoA normalized to total RhoA in HUVECs with and without knockdown of hCLOCK.

We then reconfirmed the hypoxia response by quantifying ROS production and HUVEC tube formation. A control HUVEC group in normoxic conditions was transduced with a control retroviral vector as described previously and served as a baseline for comparison of ROS levels. Two hypoxic HUVEC groups were transduced with scrambled (SCR) and hCLOCK knockdown (shCLOCK) retroviral vectors. Relative ROS levels were determined with DCFH analysis (Figure 2(b)). Tube formation was visualized with a digital microscope camera. Efficacy of hCLOCK knockdown via shCLOCK transduction is shown in Figures 2(a) and 2(d). Following transduction with the scrambled retroviral vector (SCR) in a hypoxic environment, DCFH analysis shows a statistically significant increase in relative ROS levels in HUVECs when compared to the normoxic control (*p* < 0.01, Figure 2(b)). Tube formation in this group was significantly decreased (*p* < 0.01, Figure 2(c)). Interestingly though, relative ROS levels of the cells in the hypoxic environment transduced with the hCLOCK knockdown retroviral vector (shCLOCK) resulted in ROS levels significantly less than the SCR group (*p* < 0.05, Figure 2(b)), though still higher than control levels. Relative tube formation was significantly higher than the SCR group (*p* < 0.05, Figure 2(c)). These findings directly implicate hCLOCK in the pathway of ROS production as well as the more physiologic measure of tube-formation inhibition.

### 3.3. hCLOCK Activates the RhoA and NF-*κ*B Signaling Pathways in HUVECs

**Figure 3:**
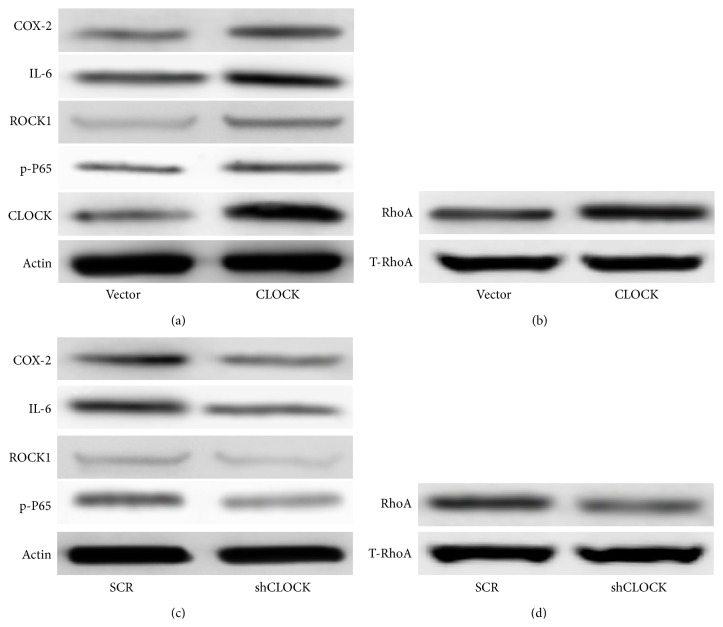
Induction of the RhoA and NF-*κ*B pathways by hCLOCK. (a) Western blot analysis of RhoA pathway effector ROCK1 and NF-*κ*B pathway effectors COX-2, IL-6, and p-P65 in HUVECs transduced with control vector or hCLOCK-overexpressing vector. Expression levels are normalized to *β*-actin. (b) Western blot analysis showing activated RhoA normalized to total RhoA in HUVECs transduced with control vector or hCLOCK-overexpressing vector. (c) Western blot analysis of the same RhoA and NF-*κ*B pathway effectors in HUVECs transduced with scrambled control vector or shCLOCK vector. (d) Western blot analysis showing activated RhoA normalized to total RhoA in HUVECs transduced with scrambled control vector or shCLOCK vector.

We performed initial testing of our hypothesis that CLOCK activates the RhoA and NF-*κ*B signaling pathways by overexpressing hCLOCK and observing downstream protein levels. Knowing that hypoxia induces ROS production, we conducted all following experiments under hypoxic conditions. Expression levels of key proteins in the RhoA and NF-*κ*B signaling pathways were measured via Western blot in HUVEC groups which were transduced with either a control retroviral vector or an hCLOCK-overexpressing retroviral vector. Protein expression levels of hCLOCK itself were observed, confirming proper technique. COX-2, IL-6, and p-P65, major effectors in the NF-*κ*B signaling pathway, were significantly upregulated in the HUVEC group transduced with the hCLOCK-overexpressing vector, compared to control (Figure 3(a)). Likewise, ROCK1 and RhoA, key proteins in the RhoA pathway, were significantly upregulated in the cell group transduced with the hCLOCK-overexpressing vector, compared to control (Figures 3(a) and 3(b)). These findings confirm that CLOCK is involved in the RhoA and NF-*κ*B signaling pathways, key pathways in tumorigenesis, and the inflammatory response.

### 3.4. Inhibition of hCLOCK Deactivates the RhoA and NF-*κ*B Signaling Pathways in HUVECs

We then sought to confirm a direct relationship between hCLOCK and the RhoA and NF-*κ*B signaling pathways. Inhibition of hCLOCK was performed to evaluate this converse association. Expression levels of the same key proteins in these pathways were measured via Western blot following transduction of HUVECs with the shCLOCK (hCLOCK knockdown) vector. These results were then compared to protein expression levels in HUVECs transduced by a scrambled control retroviral vector.

We found that all of the key effector proteins previously examined, COX-2, IL-6, p-P65, ROCK1, and RhoA, were significantly downregulated by transduction of shCLOCK, compared to control (Figures 3(c) and 3(d)), thus identifying hCLOCK as directly involved in the activation of the RhoA and NF-*κ*B signaling pathways.

### 3.5. Inhibition of RhoA Attenuates hCLOCK-Induced p-P65, ROCK1, IL-6, and COX-2 Expression but Not ROS Production

**Figure 4:**
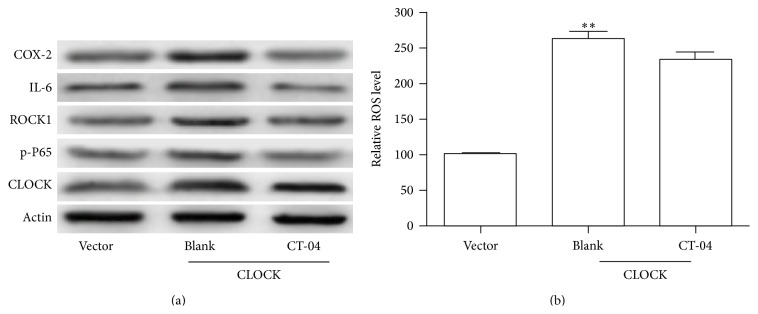
Induction of the NK-*κ*B pathway by RhoA. (a) Western blot analysis of RhoA pathway effector ROCK1 and NF-*κ*B pathway effectors COX-2, IL-6, and p-P65 in HUVECs overexpressing hCLOCK and exposed to 5 *μ*g/mL RhoA inhibitor CT-04 or control solution for 24 hours. Vector control group consists of HUVECs transduced with control retroviral vector exposed to control solution. (b) Bar graph showing relative ROS levels in the same HUVEC groups. ^*∗∗*^ *p* < 0.01 compared to vector control.

We subsequently targeted the RhoA signaling pathway to discern whether downstream effectors were suppressed with inhibition of RhoA itself. We examined HUVECs transduced with the hCLOCK-overexpressing vector, exposed to either cell-permeative Rho inhibitor C-3 transferase (CT-04) or blank solution. The control hCLOCK-overexpressing group expressed higher levels of the RhoA and NF-*κ*B pathway effectors as expected. When the hCLOCK-overexpressing HUVECs were exposed to the Rho inhibitor (CT-04) solution, however, all of the previously overexpressed effector proteins were found to be downregulated (Figure 4(a)). These findings proved that inhibition of hCLOCK attenuates the production of the RhoA and NF-*κ*B pathway effectors COX-2, IL-6, p-P65, and ROCK1. We then sought to examine the effects of hCLOCK on ROS production.

The hCLOCK-overexpressing cell group which was exposed to blank solution produced 2.5-fold more ROS than the control vector group (*p* < 0.01), as expected (Figure 4(b)). Interestingly, however, the hCLOCK-overexpressing cell group exposed to the Rho inhibitor (CT-04) produced a similar amount of ROS compared to the noninhibited group (Figure 4(b)). This interesting result shows that while inhibition of RhoA attenuates the RhoA and NF-*κ*B pathway effectors, it does not in fact suppress the production of ROS. This suggests that ROS may either have a place in the overall signaling pathway upstream of RhoA or have a separate pathway.

### 3.6. The hCLOCK-Induced RhoA and NF-*κ*B Pathways Are Inhibited in the Presence of a ROS Scavenger

**Figure 5:**
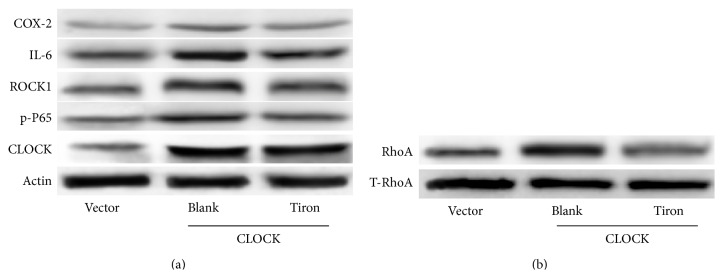
Effect of ROS levels on RhoA and NF-*κ*B pathways. (a) Western blot analysis of RhoA pathway effector ROCK1 and NF-*κ*B pathway effectors COX-2, IL-6, and p-P65 in HUVECs overexpressing hCLOCK and treated with either 10 *μ*M Tiron or control solution for 12 hours. Vector control group consists of HUVECs transduced with control retroviral vector exposed to control solution. (b) Western blot analysis of activated RhoA normalized to total RhoA in HUVECs overexpressing hCLOCK and treated with either 10 *μ*M Tiron or control solution for 12 hours. Vector control group consists of HUVECs transduced with control retroviral vector exposed to control solution.

**Figure 6:**
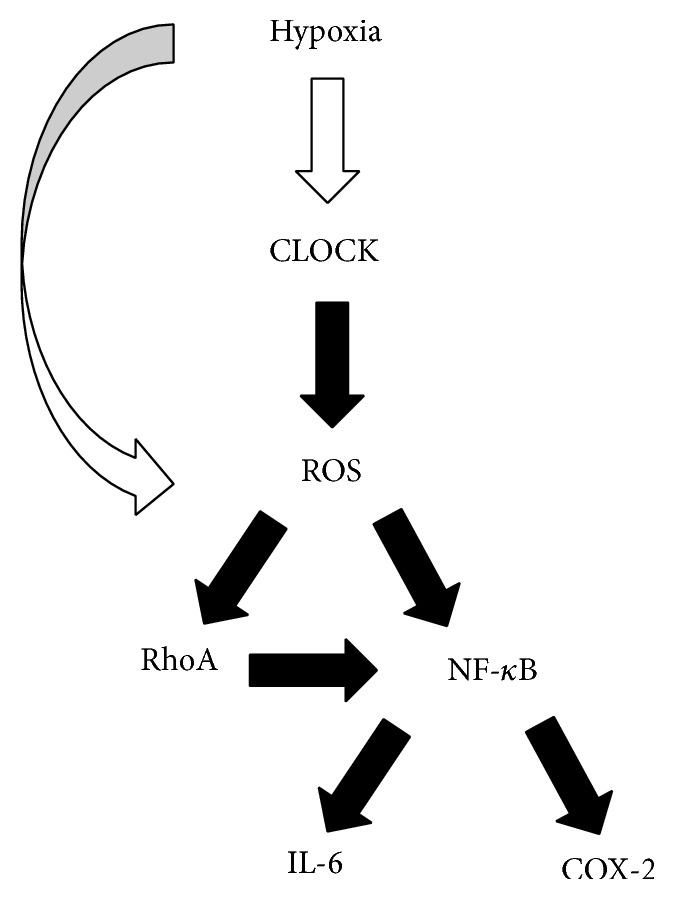
Proposed signaling pathway of the hCLOCK-induced hypoxia response. hCLOCK is the primary inducer of the hypoxia response, leading to ROS production, which in turn induces the RhoA and NF-*κ*B signaling pathways and upregulates key downstream inflammatory effectors IL-6 and COX-2.

In order to answer the question raised by the previous experiment, we decided to target ROS and reveal its role in the hypoxic response. We used Tiron, a ROS scavenger, to reduce cellular ROS levels and examine the effects on downstream proteins. We conducted this experiment with three groups: (1) HUVECs transduced with control GFP vector and treated with control solution, (2) HUVECs transduced with hCLOCK-overexpressing vector and treated with control solution, and (3) HUVECs transduced with hCLOCK-overexpressing vector and treated with Tiron solution. Similar to the prior experiment, the hCLOCK-overexpressing group treated with control solution expressed increased levels of COX-2, IL-6, p-P65, RhoA, and ROCK1 (Figures 5(a) and 5(b)). When the hCLOCK-overexpressing HUVECs were treated with Tiron solution, however, they expressed decreased protein levels of all of these downstream effectors. Our finding that decreased ROS levels inhibit the RhoA and NF-*κ*B pathways, while inhibition of RhoA inhibits these same pathways without significantly altering ROS levels, further elucidates the hCLOCK-induced hypoxic response pathway. We have determined that hypoxia increases the expression of hCLOCK, which leads to ROS production, which then activates the RhoA and NF-*κ*B pathways (Figure 6).

## 4. Discussion

We hereby describe the hCLOCK-induced hypoxia response pathway, which leads to a Rho GTPase and NF-*κ*B mediated endothelial inflammatory response. Specifically, this study finds that (1) expression levels of hCLOCK and RhoA are increased in a hypoxic state, (2) hypoxia induces ROS production via hCLOCK and inhibits HUVEC tube formation, (3) hCLOCK activates the RhoA and NF-*κ*B signaling pathways in HUVECs, (4) inhibition of hCLOCK deactivates the RhoA and NF-*κ*B signaling pathways in HUVECs, (5) inhibition of RhoA attenuates hCLOCK-induced p-P65, ROCK1, IL-6, and COX-2 expression, but not ROS production, and (6) the hCLOCK-induced RhoA and NF-*κ*B pathways are inhibited in the presence of a ROS scavenger. Briefly, hypoxia increases the expression of hCLOCK, which leads to ROS production, which then activates the RhoA and NF-*κ*B pathways.

We pursued these particular pathways on the basis of several areas of prior research linking hCLOCK with the hypoxia response, and the hypoxia and inflammatory responses with tumorigenesis. Additionally, the Rho GTPases and the NF-*κ*B pathway had both been implicated in the inflammatory response. We sought to find the intersection between these pathways.

Our initial experiments confirmed the initial link in the hCLOCK-induced hypoxia response pathway, that hypoxic conditions increase both hCLOCK and RhoA levels. All further experiments were carried out under hypoxic conditions. The hypoxic response pathways are myriad, though hCLOCK had not previously been definitively characterized.

The biochemical involvement of hCLOCK in ROS production has not previously been characterized. By knocking down hCLOCK via a shCLOCK retroviral vector, we confirmed that the hypoxic response of ROS production was mediated by hCLOCK. This resulted in a significant decrease in ROS levels. These findings suggest that although hCLOCK may not be the sole mediator of ROS production, it plays a major role and is thereby a potential target for future research or therapy.

As prior research had shown a link between the Rho GTPases and the NF-*κ*B pathways and the inflammatory response, we then turned our attention to the effectors in these pathways. RhoA and ROCK1, the major effectors in the RhoA pathway, as well as COX-2, IL-6, and p-P65, the major effectors in the NF-*κ*B pathway, were found to be upregulated by hCLOCK. These effectors of both pathways were downregulated with knockdown of hCLOCK, inhibition of RhoA, or decrease in ROS levels.

The ROS scavenger we used, Tiron, is known to also bind metals including iron, copper, uranium, vanadium, beryllium, and chromium; thus it is conceivable that off-target effects may have confounded our study [26]. Of these metals, only iron and copper would be reasonably expected in HUVECs. Copper has previously been shown to activate the NF-*κ*B pathway; however it achieves this via the production of ROS, similar to hCLOCK [27]. Our shCLOCK knockdown targeted specifically at hCLOCK causes inhibition of this pathway, demonstrating that the two inducers, copper and hCLOCK, have similar though independent roles in inducing ROS production.

The RhoA and NF-*κ*B pathways each have their own profound spheres of influence within the grand world of human biology and are brought together with hCLOCK in the hypoxic inflammatory response. To date, research has been performed on multiple links in a complex chain of biochemical pathways ultimately connecting regulators such as hCLOCK with inflammation and tumorigenesis. Our novel characterization of the hCLOCK-induced hypoxia response helps to complete this chain.

In conclusion, hCLOCK induces Rho GTPase mediated endothelial dysfunction and NF-*κ*B mediated inflammatory responses. This study provides novel insight into the hypoxia response and we hope that these findings may help further elucidate some aspects of the complex biology of inflammation and tumorigenesis.
